# Cortical Thickness and Subcortical Gray Matter Reductions in Neuropsychiatric Systemic Lupus Erythematosus

**DOI:** 10.1371/journal.pone.0009302

**Published:** 2010-03-24

**Authors:** Rex E. Jung, Judith M. Segall, Rachael G. Grazioplene, Clifford Qualls, Wilmer L. Sibbitt, Carlos A. Roldan

**Affiliations:** 1 Department of Neurosurgery, University of New Mexico, Albuquerque, New Mexico, United States of America; 2 Mind Research Network, University of New Mexico, Albuquerque, New Mexico, United States of America; 3 Department of Mathematics, University of New Mexico, Albuquerque, New Mexico, United States of America; 4 Department of Internal Medicine, University of New Mexico, Albuquerque, New Mexico, United States of America; Universidad Peruana Cayetano Heredia, Peru

## Abstract

Within systemic lupus erythematosus (SLE) patients can be divided into groups with and without central nervous system involvement, the latter being subcategorized as neuropsychiatric systemic lupus erythematosus (NPSLE). While a number of research groups have investigated NPSLE, there remains a lack of consistent application of this diagnostic criteria within neuroimaging studies. Previous neuroimaging research suggests that SLE patients have reduced subcortical and regional gray matter volumes when compared to controls, and that these group differences may be driven by SLE patients with neuropsychiatric symptoms. The current study sought to compare measures of cortical thickness and subcortical structure volume between NPSLE, SLE, and healthy controls. We hypothesized that patients with NPSLE (N = 21) would have thinner cortex and reduced subcortical gray matter volumes when compared to SLE (N = 16) and control subjects (N = 21). All subjects underwent MRI examinations on a 1.5 Tesla Siemens Sonata scanner. Anatomical reconstruction and segmentation were performed using the FreeSurfer image analysis suite. Cortical and subcortical volumes were extracted from FreeSurfer and analyzed for group differences, controlling for age. The NPSLE group exhibited decreased cortical thickness in clusters of the left frontal and parietal lobes as well as in the right parietal and occipital lobes compared to control subjects. Compared to the SLE group, the NPSLE group exhibited comparable thinning in clusters of the frontal and temporal lobes. Controlling for age, we found that between group effects for subcortical gray matter structures were significant for the thalamus (F = 3.06, p = .04), caudate nucleus (F = 3.19, p = .03), and putamen (F = 4.82, p = .005). These results clarify previous imaging work identifying cortical atrophy in a mixed SLE and NPSLE group, and suggest that neuroanatomical abnormalities are specific to SLE patients diagnosed with neuropsychiatric symptoms. Future work should help elucidate the underlying mechanisms underlying the emerging neurobiological profile seen in NPSLE, as well as clarify the apparent lack of overlap between cortical thinning and functional activation results and other findings pointing to increased functional activation during cognitive tasks.

## Introduction

Neuropsychiatric systemic lupus erythematosus (NPSLE) is highly prevalent in and increases significantly the morbidity and mortality of patients with SLE [1], [2], [3], [4]. In 1999, the American College of Rheumatology developed 19 discrete neuropsychiatric syndromes that comprised NPSLE, spanning both central (e.g., cerebrovascular disease) and peripheral (e.g., neuropathy) nervous systems [5]. This nomenclature has since been validated in several subsequent studies of prospective patient cohorts [6], [7], [8], [9], [10]. However, no “gold standard” currently exists by which to discriminate NPSLE with high sensitivity and specificity despite a combination of behavioral (e.g., cognitive dysfunction), radiological (e.g., ischemic changes), and laboratory findings (e.g., cytokine production) [11].

A number of research groups have undertaken imaging of NPSLE, with hope of providing much needed specificity of central nervous system involvement within SLE. Overt lesions and metabolic abnormalities observed with Magnetic Resonance Imaging may occur in 25 to 75% of NPSLE patients and are positively associated with disease severity, disease activity, age, and neurologic events [12], [13], [14], [15], [16], [17], [18], [19], [20], [21], [22], [23]. Lesions identified on MRI are attributed to in situ thrombosis, vasculitis, edema, hemorrhage, atherosclerosis, or atheroembolism [24]. In the first prospective study to assess MRI and postmortem tissue in NPSLE, Sibbitt et al. studied 14 subjects characterized by the ACR nomenclature and case definitions for NPSLE [24]. The findings of this study supported previous prevalence studies of brain abnormalities in NPSLE, showing small punctate focal lesions in white matter being the most common MRI finding (100%), followed by cortical atrophy (64%), ventricular dilation (57%), cerebral edema (50%), diffuse white matter abnormalities (43%), focal atrophy (36%), cerebral infarction (29%), acute leukoencephalopathy (25%), and intracranial hemorrhage (21%) [13], [14], [15], [16], [21].

Cerebral atrophy has been demonstrated repeatedly in SLE using both Computed Tomography (CT) and Magnetic Resonance Imaging (MRI). However, prevalence rates vary widely due to different imaging modalities, different patient selection criteria, and the prevalence of qualitative (as opposed to quantitative) assessments [23], [25], [26], [27], [28], [29], [30], [31], [32], [33], [34]. To date, only one study has attempted to use automated analysis methods to assess cortical atrophy in SLE [35]. This group studied eighty-nine patients diagnosed with SLE and used Voxel-Based Morphometry (VBM) to assess gray and white matter volume differences compared to forty-four healthy controls. They specifically excluded two subjects with history of stroke. Patients with active central nervous system involvement (48%) were compared to those without such involvement using the ACR guidelines [5]. Results indicated reduced gray matter volumes in SLE patients compared to controls in frontal, occipital, and temporal lobes as well as in limbic areas; patients with CNS involvement (i.e., NPSLE) were driving this relationship, as SLE patients without CNS involvement were not significantly different from controls [35].

Cortical atrophy is apparent in SLE on standard CT and MRI images, and volume differences have been demonstrated in widely distributed brain regions using automated segmentation techniques [35]. We sought to use a complementary, semi-automated, segmentation technique that accurately measures cortical thickness and specific subcortical structure volume [36], [37] as opposed to gray matter volume [38], [39], [40]. We assess group cortical thickness and subcortical gray matter volume differences between NPSLE, SLE, and age-and-gender matched healthy volunteers. As the previous VBM study did not control for age [35], we also aim to control for any contribution of age effects on cortical thickness and subcortical gray matter reductions in this cohort. We hypothesized that, after controlling for age, patients diagnosed with NPSLE would have thinner cortex and reduced subcortical gray matter volumes than patients without NPSLE or control subjects.

## Results

### Demographic data

NPSLE, SLE, and Control subjects did not differ significantly in terms of age (F = 1.31, p = 2.78; NPSLE = 39.2+/−12.5; SLE = 37.3+/−12.6; controls  = 33.2+/−11.6), gender (%, %, and % females, respectively) and premorbid intellectual functioning (F = .40, p = .67; NPSLE = 44.9+/−7.1; SLE = 46.0+/−8.0; controls  = 47.0+/−7.09).

### Clinical, laboratory and treatment features

Six NPSLE patients had past stroke (two had current stroke); twelve patients had past TIA (three had current TIA); no patients had past or current acute psychosis; four patients had past seizure disorder (four had current seizure disorder); five patients had past acute confusional state (one patient had current acute confusional state); five patients had past moderate or severe cognitive dysfunction (one patient had current moderate or severe cognitive dysfunction). Overall, twenty-one patients demonstrated either acute or past (or both acute and past) symptoms of NPSLE. Mean SLEDAI score at the time of MRI scan was 13.9 (range 3–43; SD = 11.2) for NPSLE and 9.1 (range 1–19; SD = 5.8) for SLE. At the dates of MRI, 42 % of patients were on steroid use. Mean SLICC/ACR DI scores were 4.4 (range 0–9; SD = 2.7) for NPSLE and 2.1 (range 0–4; SD = 1.2) for SLE.

### Cortical Thickness Differences

**Figure 1:**
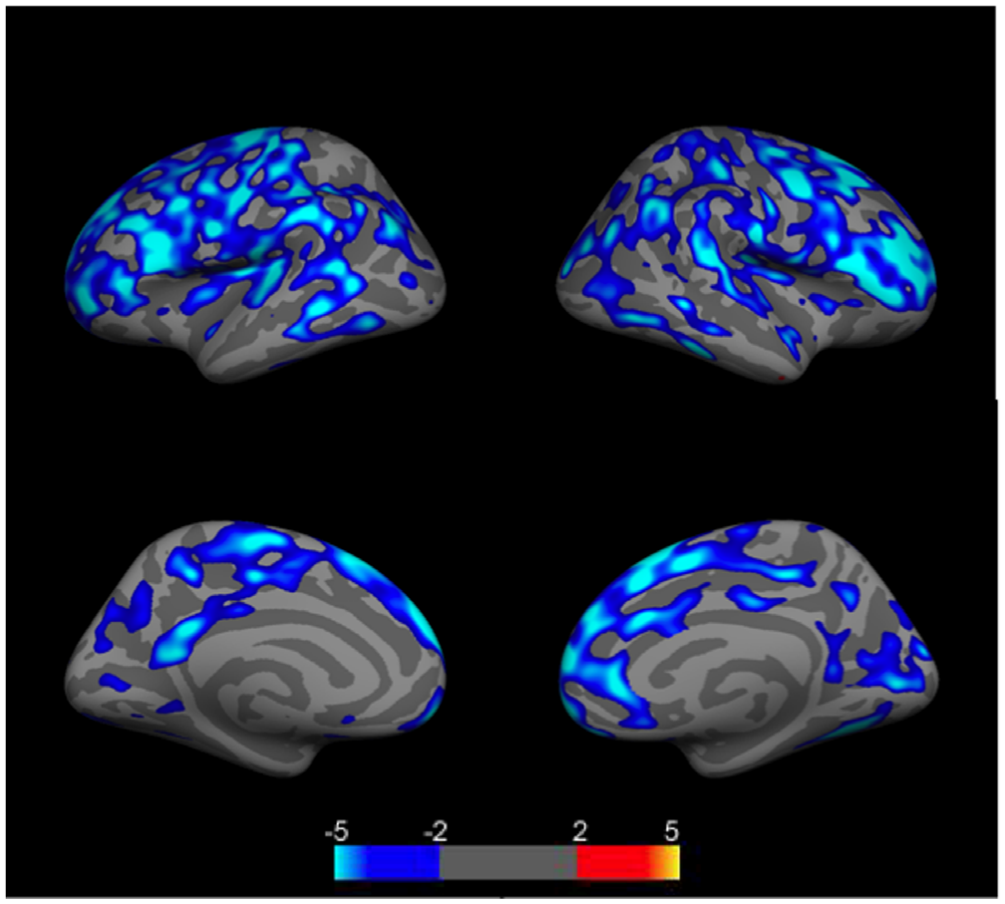
Group comparison of cortical thickness relationships to age for the entire group of NPSLE, SLE, and control subjects (N = 58). Images show clusters of lower (blue clusters) cortical thickness values related to age. Clusters are displayed in the range of p≤.01 to p≤.0001 (color scale shows −log (10) p-value). Top left  =  left lateral hemisphere; Bottom left  =  left medial hemisphere; Top right  =  right lateral hemisphere; Bottom right  =  right medial hemisphere. Light blue regions indicate regions where age and cortical thickness were significantly related (p<.05) corrected for multiple comparisons (FDR).

**Figure 2:**
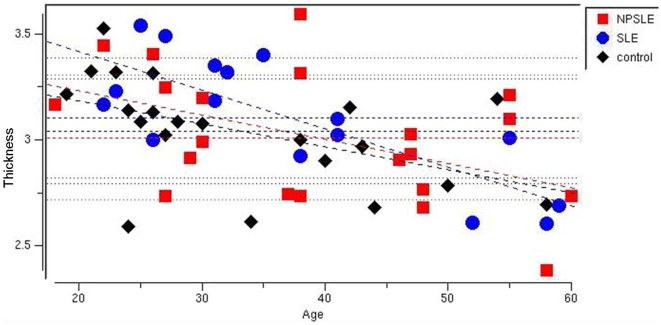
Scatterplot showing individual cortical thickness values (in millimeters) obtained from the left rostrofrontal cortex for the sample. Red squares  =  NPLSE patients; Blue circles  =  SLE patients (blue circles); Black diamonds  =  control subjects, stratified by age. All three groups show decreasing trends for cortical thickness in all significant regions (light blue in Figure 1).

Because controls were somewhat younger than either patient group, and all groups had a wide age range (18–60), we first examined the effects of age on cortical thickness across the entire sample. We found that age had a significant effect on cortical thickness across the group, with decreasing thickness associated with increasing age in numerous brain regions (p<.05 corrected for multiple comparisons using FDR)(Figure 1). There were no brain regions in which increased age was associated with increased cortical thickness. Interestingly, NPSLE, SLE, and control subjects had different slopes and intercepts in various regions where age effects were observed, suggesting an interaction between disease processes and age (Figure 2).

**Figure 3:**
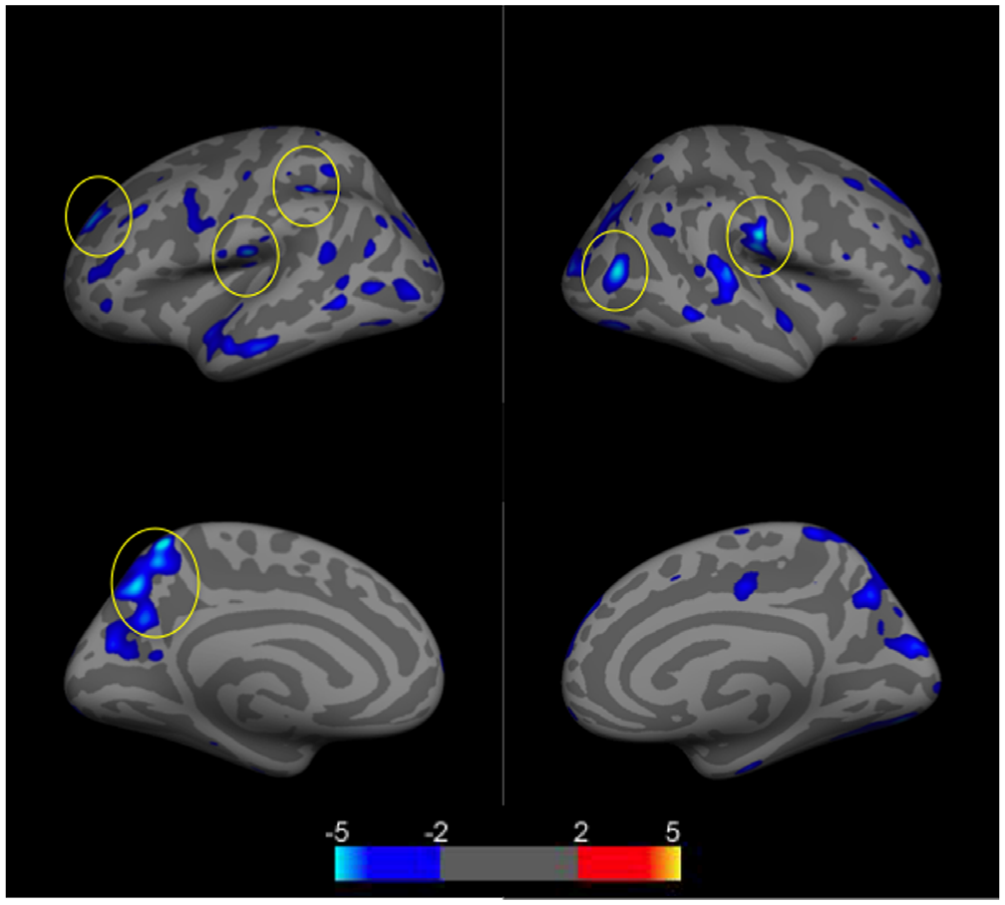
Group comparison of cortical thickness differences between NPSLE patients (N = 21) and control subjects (N = 21). Images show clusters of lower (blue clusters) cortical thickness values controlling for age. Clusters are displayed in the range of p≤.01 to p≤.0001 (color scale shows −log (10) p-value). Clusters which survived FDR correction for multiple correction (p≤.05 are encircled). Top left  =  left lateral hemisphere; Bottom left  =  left medial hemisphere; Top right  =  right lateral hemisphere; Bottom right  =  right medial hemisphere.

We next assessed cortical thickness differences between NPSLE, SLE, and control groups, controlling for age. We first assessed differences between NPSLE and controls, and found numerous regions in which NPSLE patients had thinner cortices compared to controls (p<.01 uncorrected). Regions which survived FDR statistical correction (light blue) (p<.05), included the postcentral, supramarginal, rostralmiddle frontal, and precuneus gyri in the left hemisphere, and the inferior parietal and postcentral gyri in the right hemisphere(Figure 3).

**Figure 4:**
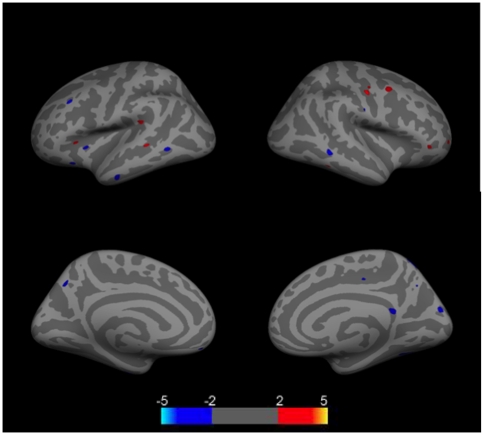
Group comparison of cortical thickness differences between SLE patients (N = 16) and control subjects (N = 21). Images show clusters of higher (red clusters) and lower (blue clusters) cortical thickness values controlling for age. Clusters are displayed in the range of p≤.01 to p≤.0001 (color scale shows −log (10) p-value). No clusters survived FDR correction for multiple correction (p≤.05). Top left  =  left lateral hemisphere; Bottom left  =  left medial hemisphere; Top right  =  right lateral hemisphere; Bottom right  =  right medial hemisphere.

When SLE patients were compared to control subjects, there were small scattered clusters in which patients had increased or decreased cortical thickness. None survived FDR correction (Figure 4).

**Figure 5:**
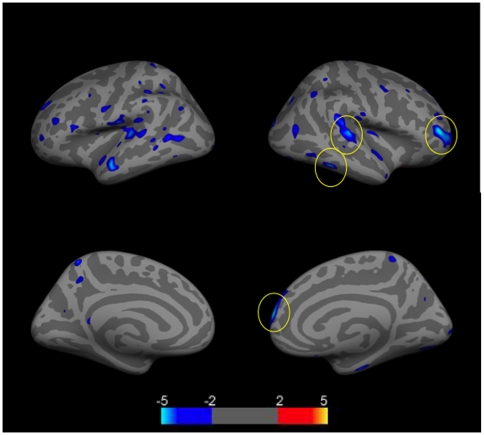
Group comparison of cortical thickness differences between SLE patients (N = 16) and NPSLE patients (N = 21). Images show clusters of lower (blue clusters) cortical thickness values controlling for age. Clusters are displayed in the range of p≤.01 to p≤.0001 (color scale shows −log (10) p-value). Clusters which survived FDR correction for multiple correction (p≤.05 are encircled). Top left  =  left lateral hemisphere; Bottom left  =  left medial hemisphere; Top right  =  right lateral hemisphere; Bottom right  =  right medial hemisphere.

We next assessed differences between NPSLE and SLE patients, and found numerous regions in which NPSLE patients had thinner cortices compared to SLE patients (p<.01 uncorrected). Regions which survived FDR statistical correction (light blue) (p<.05), included the rostralmiddle frontal, superior frontal, superior temporal, inferior temporal, and lateral occipital gyri in the right hemisphere (Figure 5). No regions survived FDR correction in the left hemisphere.

Finally, we assessed subcortical gray matter differences between groups, focusing on the hippocampus, amygdala, caudate nucleus, putamen, and thalamus. Controlling for age, we found that between group effects were significant for the thalamus (F = 3.06, p = .04), caudate nucleus (F = 3.19, p = .03), and putamen (F = 4.82, p = .005). Post hoc analyses showed that NPSLE subjects had lower volumes compared to controls within the thalamus (t = 2.53, p = .016) and putamen (t = 2.05, p = .05). No regions were significantly different between SLE and control subjects in post hoc analyses.

## Discussion

We found that young patients with NPSLE had lower cortical thickness in several regions of the brain compared to both SLE patients without past or current NPSLE and normal controls. These results clarify previous imaging studies showing cortical atrophy in SLE [23], [24], [25], [26], [27], [28], [30], [31], [32], [33], [34], [41], [42], [43]. Indeed, the only study using automated segmentation techniques noted that “no difference between SLE patients without CNS involvement and healthy controls was observed” [35]. This conforms well with our data showing significant gray matter reductions in NPSLE, but not SLE, compared to control subjects. However, our study suggests that cortical thickness on MRI separates patients with NPSLE from those without NPSLE and healthy controls.

The regions in which we observed significant reductions of gray matter in NPSLE compared to control subjects were within the postcentral, supramarginal, rostralmiddle frontal, and precuneus gyri in the left hemisphere, and the inferior parietal and postcentral gyri in the right hemisphere. Regions in which NPSLE patients differed from SLE patients included the rostralmiddle frontal, superior frontal, superior temporal, inferior temporal, and lateral occipital gyri in the right hemisphere and there was no overlap in regions where NPSLE differed from SLE or controls. In comparing the regions in which NPSLE differed from controls, there appears to be commonality with those identified by Appenzeller et al., [34], particularly the left precuneus. The precuneus has been implicated in visuo-spatial imagery, episodic memory retrieval, perspective taking, and the experience of “agency” [44]. Moreover, the precuneus has rich cortical and subcortical connections [45] with other regions found to have reduced cortical thickness in NPSLE including the thalamus [46], inferior parietal lobule (i.e., supramarginal gyrus), and postcentral gyri.

Subcortically, we found significantly reduced gray matter volume in the thalamus, caudate, and putamen in NPSLE. the thalamus of which was also found to be reduced in volume in the prior VBM study [35]. Other studies have shown thalamus abnormalities with measures of fractional anisotropy [47] and glucose metabolism [48] in patients diagnosed with NPSLE and SLE respectively. We did not find significant hippocampal size reductions in our sample of NPSLE and SLE patients, as has been previously reported [49], although our sample size is significantly smaller than that reporting differences. However, our data suggests that NPSLE patients had smaller hippocampi than SLE patients, who had smaller hippocampi than controls. Futures studies with larger samples should elicit significant differences as demonstrated previously.

One significant difference between our study and the previous VBM study was that ours included NPSLE patients suffering from past or current cerebrovascular accident. Other imaging studies in SLE have included patients with stroke [18], [27], [30], [32], and there is significant overlap between patients with antiphospholipid syndrome, stroke, and epileptic seizures [50]. As the incidence of stroke in SLE has been found to be increased by a factor of 2.29 in a population cohort [51], exclusion of these subjects in neuroimaging studies would appear to limit generalizability of a particularly debilitating neurological consequence of disease status.

Strengths of the current study include: 1) the relatively large patient cohort, 2) the relative youth (</ = 60 years) of the patient cohort compared to previous studies, 3) whole brain as compared to ROI analyses, 4) ease, reproducibility, and automaticity of FreeSurfer methodology, and 5) the lack of differences between SLE patients without NPSLE and controls strengthen the specificity of our findings. A limitation of the study is the lack of repeated measures of the acute NPSLE patients as they progress through the acute phase of their disease. This would help to establish whether therapy or disease characteristics predominated over time in determining cortical and subcortical gray matter thickness reductions. However, these extremely ill patients are difficult to study repetitively in the acute setting, and these data would have likely increased the differences between acute NPSLE as compared to SLE patients or healthy controls. Future studies will determine if patients can be subcategorized into more tractable groups amenable to sensitive neuroimaging studies.

These results suggest that great care is needed when selecting NPSLE patients to participate in neuroimaging studies. Patients with NPSLE appear to have different cortical and subcortical gray matter thinning characteristics than those SLE patients without acute or past NPSLE symptoms (e.g., seizure, transient ischemic attack, acute confusion). It is important to include NPSLE patients in studies (including those with stroke), as well as to determine the brain mechanisms involved in order to better target therapeutic approaches designed to reduce mortality and morbidity.

What functional significance might these volume reductions have for NPSLE? Previous functional studies have demonstrated reduced glucose metabolism [52], yet increased functional activation on motor [53] and working memory tasks [54] in NPSLE. Researchers have hypothesized “increased neural recruitment” in patient cohorts in which equivalent behavioral performance is observed, in the presence of disease, designed to limit the functional impact of neuronal injury [55]. Other studies in normal cohorts of children have found decreased cortical thickness associated with increased functional activations [56], [57]. Indeed, some of the regions in which increased “neural recruitment” were observed during functional imaging studies included the precuneus and putamen [53] and inferior parietal and frontal polar regions [54]: similar regions in which our study found decreased thickness in NPSLE subjects compared to controls. While quite preliminary in nature, these findings add to an emerging body of literature suggesting that brain structure and function might not increase and decrease in tandem, but might reflect a complex interplay of excitatory and inhibitory networks designed to optimize functional capacity while consuming minimal resources [58]. Future research in NPSLE presents a particularly fruitful patient group by which various hypotheses' regarding the complex interplay of brain structure and function at various stages of disease might be undertaken.

## Methods

### Sample

This study was conducted according to the principles expressed in the Declaration of Helsinki, and was approved by the Institutional Review Board of the University of New Mexico. All subjects provided written informed consent for the collection of samples and subsequent analysis. The sample consisted of thirty-seven SLE patients, recruited from the Rheumatology Clinics of the University of New Mexico, ranging in age from 18 to 60 (35 females). All subjects were diagnosed with SLE based on the 1997 update to the 1982 American College of Rheumatology Revised Criteria for Classification of Systemic Lupus Erythematosus [59]. Twenty-one of the SLE patients had major NPSLE defined as current or past stroke or transient ischemic attack (TIA), current or past confusional state, moderate or severe cognitive dysfunction, current or past seizures, or current or past psychosis. Sixteen patients had SLE, but no current or past NPSLE.

These 2 groups of patients were compared to twenty-one healthy controls (18 females). Participants were screened for conditions that would prohibit undergoing an MRI scan (e.g., metal implant, orthodontic braces, severe claustrophobia).

### Clinical Measures

The Systemic Lupus Erythematosus Disease Activity Index (SLEDAI) [60] and Systematic Lupus International Collaborating Clinics/America College of Rheumatology Damage Index (SLICC/ACR DI) [61] were administered by an experienced rheumatologist (WLS) to each patient. Clinical diagnosis of NPSLE was defined by presence of past or current: stroke, transient ischemic accident, psychosis, seizure disorder, confusional state, and/or moderate or severe cognitive dysfunction. No SLE patients had any past or current evidence of any of these clinical diagnoses.

### Behavioral Measure

The Wide Range Achievement Test – 3^rd^ Revision (WRAT-3) Reading subtest, was used as a measure of premorbid cognitive functioning. This measure requires subjects to read single words with irregular phonetic spelling (e.g., colonel), and has been found to be resistant to the effects of cognitive decline due to neurological or psychiatric disease [62].

### Image Acquisition and Processing

MR examinations were performed on a 1.5T Siemens Sonata scanner using an 8-channel phased array head coil. Structural imaging was obtained using a T1 coronal gradient echo sequence [TE = 4.76 ms; TR = 12 ms; Voxel Size  = 1×1×1.5 mm; acquisition time  = 7:15]. Subjects' heads were stabilized with tape across the forehead and padding around the sides. For all scans, each T1 was reviewed for image quality. Cortical reconstruction and volumetric segmentation were performed with the FreeSurfer image analysis suite, which is documented and freely available for download online (http://surfer.nmr.mgh.harvard.edu/). The methodology for FreeSurfer is described in full in several papers [36], [37], [63], [64], [65], [66], [67], [68], [69], [70], [71]. For this paper, we are focused on the cortical thickness results and volumes of select subcortical structures. Procedures for the measurement of cortical thickness have been validated against histological analysis [72] and manual measurements [73], [74]. The results of the automatic segmentations were reviewed and any errors were corrected.

### Statistical Analysis

To investigate the correlation between cortical thickness measurements between groups (NPSLE, SLE, controls) we performed a surface-based group analysis using tools within FreeSurfer. First, the subjects' surface was smoothed using a full-width/half-maximum Gaussian kernel of 10 mm. This smoothing was done so that all subjects in this study could be displayed on a common template (an average brain as described at http://surfer.nmr.mgh.harvard.edu/) in order to perform and visualize a group analysis. Freesurfer's mri glmfit was used to fit a general linear model at each vertex in the cortex to perform between group averaging and statistical inference on the cortical surface. The design matrix consisted of three discrete groups, (NPSLE, SLE, control), with age as a covariate; the slope used was different offset/intercept, different slope (DODS). The contrast matrix used investigated the average differences between cortical thickness, while regressing out the effect of age, which was a two-tailed t-test, with (NPSLE<SLE<control). We used similar statistical methods that were used in prior thickness studies [58], [75], [76], [77] to ascertain surface-based group differences using the general linear model tools within FreeSurfer. In order to correct for multiple comparisons, we used False Discovery Rate (FDR) at p<.05. We also show significant clusters at p<.01 uncorrected for hypothesis generation in this initial study. Finally, we assessed volumetric differences between groups for subcortical structural regions including the hippocampus, amygdala, thalamus, caudate nucleus, and putamen. Volumes were extracted from FreeSurfer and analyzed with SPSS 13.0 using an ANCOVA controlling for age. Group differences were assessed between NPSLE, SLE, and controls using two-tail tests. Post hoc t-tests were used to determine specific differences between groups (e.g., NPSLE vs. controls; SLE vs. controls; NPSLE vs. SLE).
